# Supplementary figures and images for: Meta-analysis of variable-temperature PCR technique performance for diagnosising Schistosoma japonicum infections in humans in endemic areas
Source: PLoS Negl Trop Dis. 2022 Jan 14;16(1):e0010136. doi: 10.1371/journal.pntd.0010136 (PMC8794272; doi:10.1371/journal.pntd.0010136)

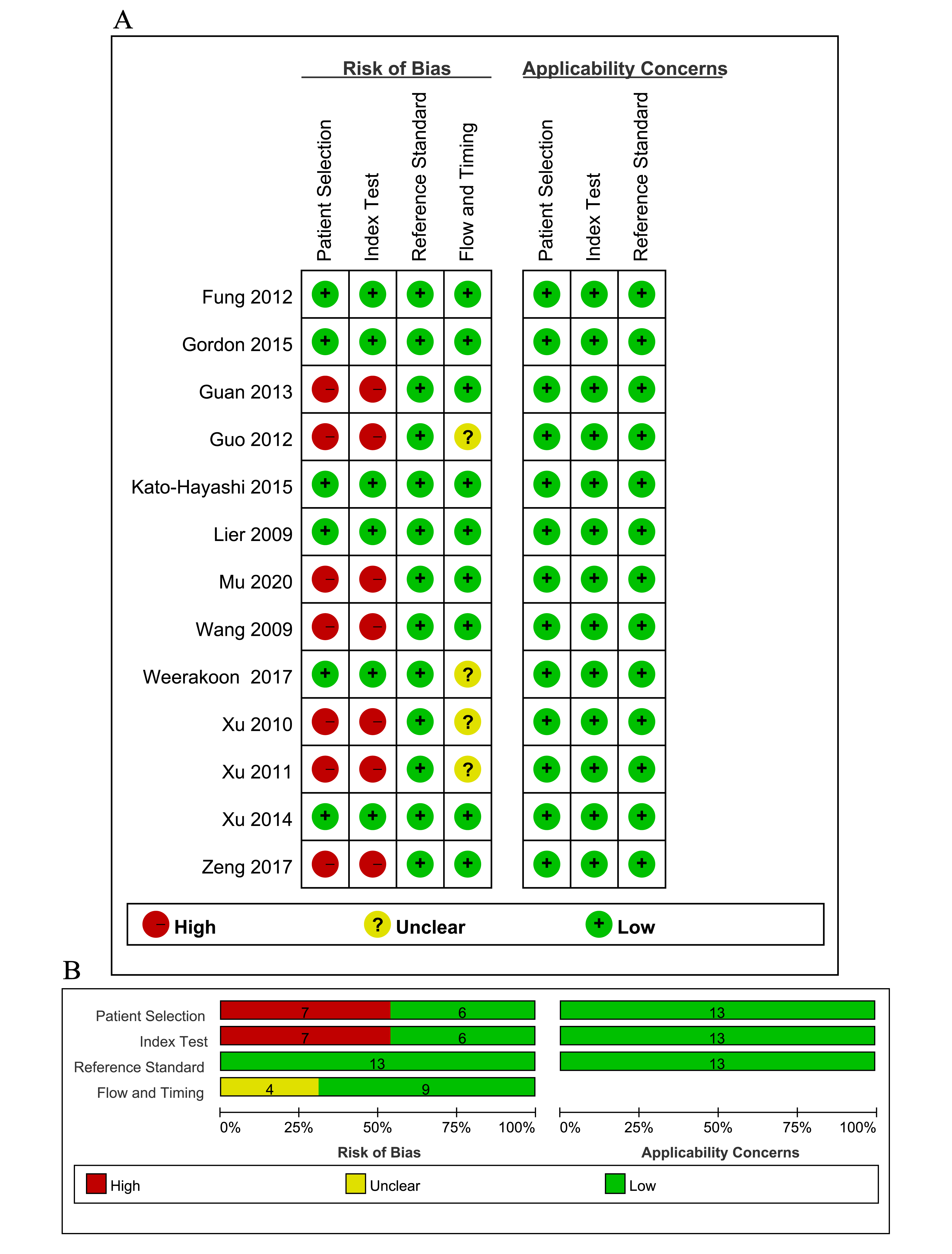

Supplement: S1 Fig — Risk of bias and applicability concerns summary (A) and graph (B). (TIF) [file pntd.0010136.s002.tif]
