# Supplementary material for: Meta-analysis of variable-temperature PCR technique performance for diagnosising Schistosoma japonicum infections in humans in endemic areas
Source: PLoS Negl Trop Dis. 2022 Jan 14;16(1):e0010136. doi: 10.1371/journal.pntd.0010136 (PMC8794272; doi:10.1371/journal.pntd.0010136)
Supplement: Attachment — Submitted filename: Reviewer report.docx [file pntd.0010136.s003.docx]

**Reviewer report**

The manuscript addresses the evaluation of molecular diagnostic technique in titled “**Meta-analysis of variable -temperature PCR technique performance for *Schistosoma japonicum* infections in human”**. Performance evaluation of sensitive diagnostic techniques such as PCR is very relevant in the area where there is a low prevalence of *S. japonicum* infection which is unable to detect even using the gold standard Kato-Katz techniques. The manuscript is original, relevant and well organized to be publishable with slight modifications.

**Minor comments**

The title is not geographically delimited but in the result section **lines, 210 & 211** indicated that all studies were conducted in schistosomiasis endemic areas of China or the Philippines. If all the data included in the analysis were from these two countries, it may need some modification of the title.

**Abstract:**

**Results:** the title is about PCR technique performance. Which PCR approach performed best and which one is poorly performed? I suggest a subgroup analysis by PCR approaches.

**Conclusion:** if you conduct subgroup analysis by PCR methods, your conclusions may also be modified.

**Keywords**: the keyword shall be arranged alphabetically.

**Introduction:** It will be nice if you included some introductory information about some of the PCR approaches being used for the detection of *S. japonicum*.

**Results:**

**Line 2-12-213:** It is indicated that different PCR techniques were used among the 13 studies included in the meta-analysis. It will be nice if you compare the results of the different PCR approaches (traditional, two droplets digital, real-time and nested PCR). Which PCR approaches have better sensitivity, specificity, PLR, NLR and DOR? In other words, you may conduct a subgroup analysis by PCR methods. This information helps you to forward better recommendation in the types of diagnostic method shall be used in low prevalence areas.

**Table 1:** You have to add a key at the bottom of the table indicating Tp, Ep, Fn and Tn.

**Fig 1and 2** are not visible for the readers. It needs some modifications.

**Conclusion:** If you perform subgroup analysis by types of PCR techniques, your conclusion might be modified. As you recommended blood-based PCR is better than stool-based PCR approaches.

**List of abbreviations:** several abbreviations are available in the manuscript but lack the list of abbreviations. It will be nice if you include the list of abbreviations.
