# Supplementary material for: Meta-analysis of variable-temperature PCR technique performance for diagnosising Schistosoma japonicum infections in humans in endemic areas
Source: PLoS Negl Trop Dis. 2022 Jan 14;16(1):e0010136. doi: 10.1371/journal.pntd.0010136 (PMC8794272; doi:10.1371/journal.pntd.0010136)
Supplement: Attachment — Submitted filename: Responses to reviewers.docx [file pntd.0010136.s004.docx]

In addition to the referees comments below, there is a need to proof read the manuscript for English grammar as there are quite a lot of grammatical errors throughout. As one example to give you an idea of what is required : eg lines 110-111 it states "However, it has been proved to be low sensitive in people...." Grammatically this should be "However, it has been proven to be of low sensitivity in people....."

Many thanks for pointing this out and this has now been reviewed in depth by all authors and we feel that it is now greatly improved.

Methods

Reviewer #1: Accepted. The authors have also searched Chinese databases looking for studies to include, and that is an advantage. The selection criteria and selection process is properly described, and so is the statistical methods used.

Reviewer #2: Yes

Thank you both very much for your positive comments and encouragement.

Results

Reviewer #1: Accepted. Two minor suggestions; the phrases ‘traditional PCR’ and ‘conventional PCR’ are both used in the text and figures in the meaning of non-real time PCR. The same phrase should be used throughout, preferably ‘conventional PCR’.

Thank you for highlighting these inconsistencies. We have now changed these all to‘conventional PCR’ throughout the manuscript.

In Table 1 the abbreviations Tp, Fp, Fn and Tn must be explained somewhere, i.e. written out in full.

Thank you for highlighting this omission in the Table 1 legend. We have added a note to list the abbreviations in full.

Reviewer #2: The quality of the figures (Fig 1 and 2) are not clearly visible. It needs some modification.

We have modified Figures 1 and 2 to make them clearer. In addition, we have checked their quality by uploading them to the Preflight Analysis and Conversion Engine (PACE) digital diagnostic tool. Please see the revised figures.

Conclusions

Reviewer #1: Accepted. Studies that look at diagnostic methods for *Schistosoma* infections has a challenge in that there is a lack of a good reference method with high sensitivity and specificity to which you can compare a ‘new’ test. When you compare a ‘new’ test (such as PCR), which might be a more accurate test, with a ‘poor’ reference method (with low sensitivity or specificity) the perceived performance of the ‘new’ test would be worse than the ‘true’ performance. Whether you for instance choose microscopy or antibodies as reference method might have a huge impact. Suggest a short mentioning/discussion of this.

Thank you very much for your positive comments and useful suggestions. Yes, you are right. The true performance of a PCR technique could be underestimated due to the reference standard Kata-Katz. We have added a short discussion on this point, including the importance of using latent class analyses on these data where no gold standard exists. Please see the end of the third paragraph in the discussion

The phrase ‘The common serological methods, e.g., based on the soluble antigen of eggs have also been proven to be useless.’ This might be a bit too categorical statement that is not fully supported by the reference. Suggest a slight moderation.

Agreed and modified. Please see the 5^th^ paragraph in the discussion.

The authors suggest using blood-based PCR in stead of stool, mainly for practical reasons/higher compliance. This is true, but some blood-based *Schistosoma* PCR methods has been shown to be positive for a long time after treatment. Would that be of any consequence?

Thank you very much for your good suggestions. Xu and colleges [Xu, *et al*. 2013] reported that based on rabbit-schistosome models, *S. japonicum* DNA in host serum primarily comes from the residual body of dead schistosomula and/or tegument shedding of worms within the first four weeks post-single sex schistosome infection; whereas during the spawning stage of the female schistosome, the parasite DNA in serum mainly comes from the disintegration of inactive eggs. The time period from treatment to total elimination of worm-originated DNA in serum did not last more than three weeks; whereas the DNA release from inactive eggs can last for more than 16 weeks. Therefore, there might be some influence and, as suggested, we have added a short explanation in the discussion.

Reference

Xu J, Liu AP, Guo JJ, Wang B, Qiu SJ, Sun H, Guan W, Zhu XQ, Xia CM, Wu ZD: The sources and metabolic dynamics of Schistosoma japonicum DNA in serum of the host. Parasitol Res 2013, 112(1):129-133.

Editorial and Data Presentation Modifications?

Reviewer #1: The term 'highly sensitive genes' is being used. Suggest using 'highly sensitive PCR targets' or 'gene targets' as genes themselves are not 'sensitive'.

Agreed and amended throughout. Please see the final lines of the abstract, authors’ summary and discussion.

Suggest rephrasing a few sentences I find complicated or unclear:

Line 309; we have proposed that single-sex schistosome infections in humans should be more common.. Use 'could'?

Agreed and amended. Please see line 369 (start of the 5^th^ paragraph of the discussion).

Line 316: Further research on the development of the detection is wanted as we would know what prevalence of such infections in humans or other hosts could be, if such methods are available. Complicated/unclear. Rephrase?

Agreed and amended. Please see the first paragraph on page 19 (line 375)

Summary and General Comments

Reviewer #1: This study is of interest because the epidemiological situation in many of the S japonicum-endemic areas, with low prevalence and low intensity infections, makes it necessary to look into new and improved diagnostics. The results are not novel per se, but the authors do a good job by collecting and analysing the different studies, including from Chinese databases. One challenge with this meta-analysis, also commented by the authors, is the heterogenicity of the included studies in terms of study design, PCR targets/methods etc. This makes it more difficult to draw conclusions.

Thank you very much for your positive and critical comments.

A reader who is interested in diagnostics for *S. japonicum* might not be familiar with the statistical methods for meta-analysis used in this study. That you might argue is a problem for the reader, not the authors.

Thank you very much for highlighting this. We have attempted to make these clearer for non specialist readers and hope that this is now as clear as possible.

Reviewer #2: Reviewer report

The manuscript addresses the evaluation of molecular diagnostic technique in titled “Meta-analysis of variable -temperature PCR technique performance for *Schistosoma japonicum* infections in human”. Performance evaluation of sensitive diagnostic techniques such as PCR is very relevant in the area where there is a low prevalence of *S. japonicum* infection which is unable to detect even using the gold standard Kato-Katz techniques. The manuscript is original, relevant and well organized to be publishable with slight modifications.

Thank you very much for your positive comments and encouragement.

Minor comments

The title is not geographically delimited but in the result section lines, 210 & 211 indicated that all studies were conducted in schistosomiasis endemic areas of China or the Philippines. If all the data included in the analysis were from these two countries, it may need some modification of the title.

Agreed and amended. *S. japonicum* is now only endemic in China and the Philippines so please see an amended title. We have also highlighted this further in the abstract and introduction.

Abstract:

Results: the title is about PCR technique performance. Which PCR approach performed best and which one is poorly performed? I suggest a subgroup analysis by PCR approaches.

Thank you very much for this great suggestion. We have therefore now conducted subgroup analyses based on PCR types. Please see line final sentence of the methods, Figure 6 E - G in the results, and the end of paragraph four in the discussion.

Conclusion: if you conduct subgroup analysis by PCR methods, your conclusions may also be modified.

Agreed and amended. Please see the end of paragraph four in the discussion and our conclusions

Keywords: the keyword shall be arranged alphabetically.

Agreed and amended. We have also added the endemic countries to these key words

Introduction: It will be nice if you included some introductory information about some of the PCR approaches being used for the detection of *S. japonicum*.

Agreed and added. See third paragraph of the introduction.

Results:

Line 212-213: It is indicated that different PCR techniques were used among the 13 studies included in the meta-analysis. It will be nice if you compare the results of the different PCR approaches (traditional, two droplets digital, real-time and nested PCR). Which PCR approaches have better sensitivity, specificity, PLR, NLR and DOR? In other words, you may conduct a subgroup analysis by PCR methods. This information helps you to forward better recommendation in the types of diagnostic method shall be used in low prevalence areas.

Agreed. As above, we have further conducted subgroup analyses as suggested.

Table 1: You have to add a key at the bottom of the table indicating Tp, Ep, Fn and Tn.

As above, we have now added these to Table 1.

Fig 1and 2 are not visible for the readers. It needs some modifications.

Agreed and amended. Please see the revised figures.

Conclusion: If you perform subgroup analysis by types of PCR techniques, your conclusion might be modified. As you recommended blood-based PCR is better than stool-based PCR approaches.

Agreed and added as mentioned above.

List of abbreviations: several abbreviations are available in the manuscript but lack the list of abbreviations. It will be nice if you include the list of abbreviations.

Answers: Agreed and added after author contributions.
